# Temporary impact on medical system and effectiveness of mitigation strategies after COVID-19 policy adjustment in China: a modeling study

**DOI:** 10.3389/fpubh.2023.1259084

**Published:** 2023-12-01

**Authors:** Chitin Hon, Jingyi Liang, Ruihan Chen, Zhijie Lin, Yangqianxi Wang, Wei He, Ruibin Liu, Jiaxi Sun, Qianyin Li, Lixi Liang, Minyi Zhang, Zichen Chang, Yinqiu Guo, Wenting Zeng, Tie Liu, Arlindo L. Oliveira

**Affiliations:** ^1^Department of Engineering Science, Faculty of Innovation Engineering, Macau University of Science and Technology, Taipa, Macao SAR, China; ^2^Guangzhou Laboratory, Guangzhou, Guangdong, China; ^3^Respiratory Disease AI Laboratory on Epidemic and Medical Big Data Instrument Applications, Faculty of Innovation Engineering, Macau University of Science and Technology, Taipa, China; ^4^Guangzhou Key Laboratory for Clinical Rapid Diagnosis and Early Warning of Infectious Diseases, KingMed School of Laboratory Medicine, Guangzhou Medical University, Guangzhou, China; ^5^State Key Laboratory of Respiratory Disease, National Clinical Research Center for Respiratory Disease, Guangzhou Institute of Respiratory Health, The First Affiliated Hospital of Guangzhou Medical University, Guangzhou, Guangdong, China; ^6^Instituto de Engenharia de Sistemas e Computadores: Investigação e Desenvolvimento em Lisboa, Lisboa, Portugal; ^7^Instituto Superior Técnico, Universidade de Lisboa, Lisboa, Portugal

**Keywords:** epidemic control, infectious diseases, COVID-19, vaccine, medical rescue

## Abstract

**Background:**

As China amends its “zero COVID” strategy, a sudden increase in the number of infections may overwhelm medical resources and its impact has not been quantified. Specific mitigation strategies are needed to minimize disruption to the healthcare system and to prepare for the next possible epidemic in advance.

**Method:**

We develop a stochastic compartmental model to project the burden on the medical system (that is, the number of fever clinic visits and admission beds) of China after adjustment to COVID-19 policy, which considers the epidemiological characteristics of the Omicron variant, age composition of the population, and vaccine effectiveness against infection and severe COVD-19. We also estimate the effect of four-dose vaccinations (heterologous and homologous), antipyretic drug supply, non-pharmacological interventions (NPIs), and triage treatment on mitigating the domestic infection peak.

**Result:**

As to the impact on the medical system, this epidemic is projected to result in 398.02 million fever clinic visits and 16.58 million hospitalizations, and the disruption period on the healthcare system is 18 and 30 days, respectively. Antipyretic drug supply and booster vaccination could reduce the burden on emergency visits and hospitalization, respectively, while neither of them could not reduce to the current capacity. The synergy of several different strategies suggests that increasing the heterologous booster vaccination rate for older adult to over 90% is a key measure to alleviate the bed burden for respiratory diseases on the basis of expanded healthcare resource allocation.

**Conclusion:**

The Omicron epidemic followed the adjustment to COVID-19 policy overloading many local health systems across the country at the end of 2022. The combined effect of vaccination, antipyretic drug supply, triage treatment, and PHSMs could prevent overwhelming medical resources.

## Introduction

As one of the seven human coronaviruses (HCoVs) detected to date (1), the SARS-CoV-2 genome encodes seven accessory proteins that may contribute to immune evasion (2), and ORF9c and ORF10 play key roles in viral replication and immune evasion processes (3). In the past 3 years, a dynamic zero COVID-19 strategy has been adopted in China and the number of infections, morbidities, serious illnesses, and deaths in the country have remained at a low level (4). Based on the virulence and transmission characteristics of the Omicron variant, China issued adjustments to epidemic measures on 11 November and 7 December 2022, respectively, including canceling nationwide nucleic acid tests, no longer checking health codes in cross-regional floating personnel, and isolating asymptomatic and mild illness at home (5, 6). With the policy lifted, China may have several waves of peak infections, coupled with the massive population floating and the increased immune escape ability of the SARS-CoV-2 variants (7, 8).

It has been shown that a quarter of deaths could be attributed to a shortage of healthcare resources (9). Statistically, the first wave of the Omicron epidemic in both Taiwan and Hong Kong peaked within 2 months, during which the number of infections spiked, triggering severe medical challenges on medical sources and leading to a significant increase in mortality (10, 11). When the capacity of the healthcare system is exceeded, a sudden increase in the number of infections may result in unnecessary medical demands and run-on healthcare resources. Therefore, it is particularly important to take measures early to delay the peak of the pandemic and reduce the number of patients, avoiding catastrophic medical resource challenges.

In response to the peak in infections caused by COVID-19, the key to reducing medical crowding is to decrease the proportion of critically ill patients and then minimize the number of mild and general patients. Vaccination is currently an important means of preventing severe cases and death from COVID-19 (12). A case–control study pointed out that one or two booster doses of vaccination have increased VE against medically attended COVID-19 illness (13), and receiving anti-SARS-CoV-2 vaccines effectively protected HCWs from breakthrough infections (14). As of 13 December 2022, 90.37% of the Chinese population has completed vaccination, but those over 60 and 80 years old are only 86.6 and 66.4%, respectively (15). A real-world study in Hong Kong showed that three doses of homologous inactivated vaccine decreased hospitalization and mortality well, but the utility of protection against infection was less satisfactory (16). However, the heterologous booster vaccine could reduce susceptibility to Omicron infection by 39% within 60 days (17), and it may be more helpful in infection peak mitigation. At the same time, a hierarchical medical system plays a crucial role in triaging infection cases. For example, Singapore has implemented the Public Health Preparedness Clinic (PHPC) program to strengthen the surveillance and treatment of outbreaks in the primary care system, focusing efficiently on serious and critical illnesses in general hospitals (18). In addition, adequate rapid antigen testing (RAT) and drug stockpiles facilitate a reasonable assessment of the health status of patients, allow asymptomatic and mild patients to be isolated at home, and reduce the panic of the population at the peak of infection (19).

Therefore, we aimed to simulate the likely demand for fever clinic visits and hospitalization beds in 120 days after policy liberalization and match with realistic capacity in China to assess the risk of challenges on medical resources. To determine how China can safely transition from a zero-COVID approach, we estimate the effect of four-dose vaccinations, adequate antipyretic drug supply, non-pharmacological interventions (NPIs), and triage treatment on mitigating the infection peak.

## Materials and methods

We conducted a modeling study to project the demand for medical sources (fever clinic visits and hospitalization beds, respectively) after adjustment to the COVID-19 policy in China. This modeling study relies on publicly available aggregated data only. As such, institutional review and informed consent are waived by the Institutional Review Board of the first affiliated hospital of Guangzhou Medical University (Guangzhou, China).

### Data modeling

**Figure 1:**
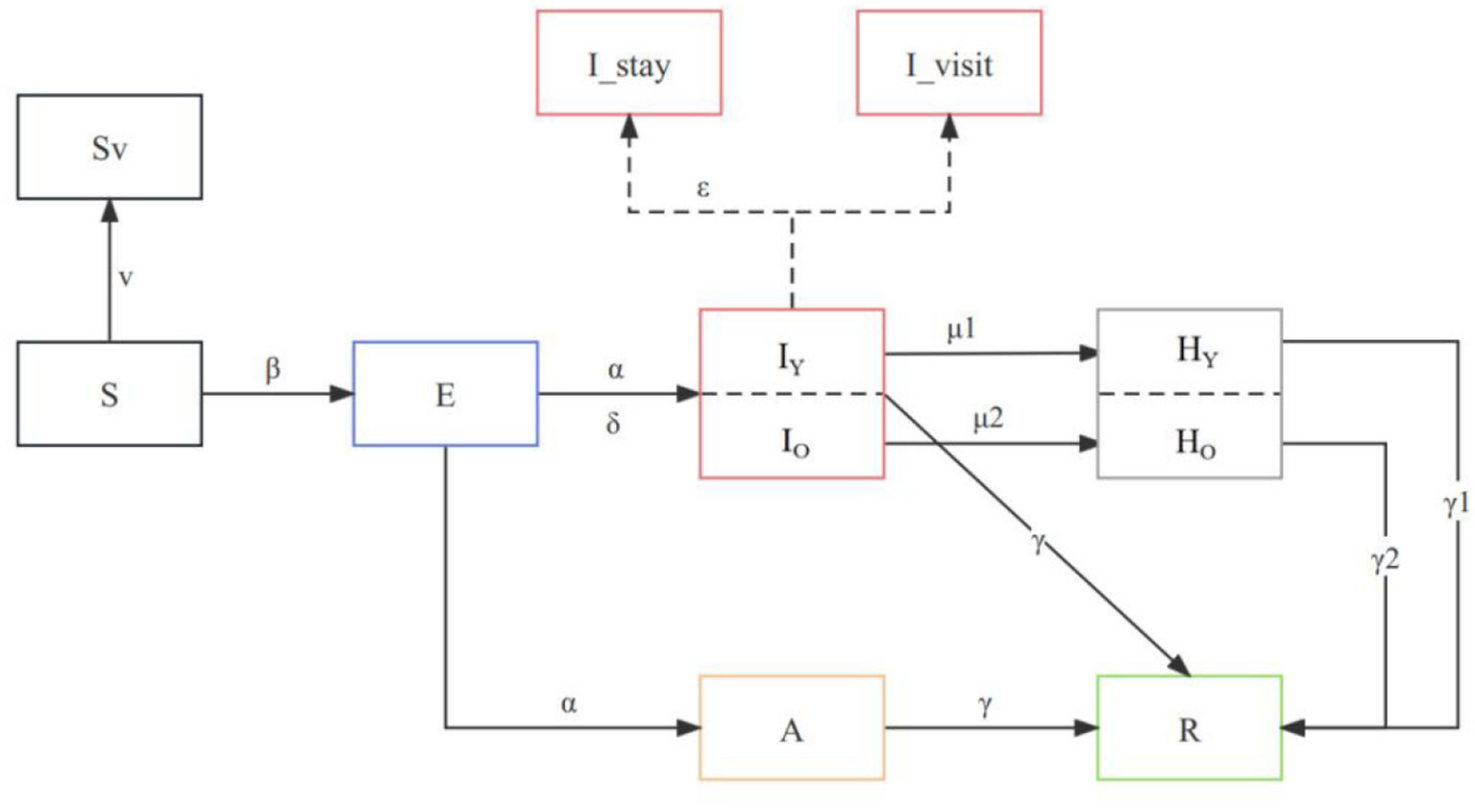
Model structure flow diagram with transition rates between states. The subscript “o” refers to the compartment of >60 years old and “y” to those who are <60 years old, and the parameters are age-specific. All compartments and parameters are defined in Supplementary Table 1.

The compartmental model was chosen to simulate the transmission of COVID-19 between populations and to explore the impact of different interventions. Transitions between compartments are simulated through a stochastic chain binomial process. We developed an age-specific stochastic compartmental model (20–22) to describe the spread of the Omicron outbreak within a population that is stratified by different states. Considering the epidemiologic history of COVID-19, we divided the population into several compartments: susceptible (S), exposed (E), asymptomatic infected (A), symptomatic infected (I), recovered (R), critical (H), and dead (D). In this study, we model the dynamics of COVID-19 transmission with the Omicron BA.5 variant by considering virus-specific parameters such as the basic reproduction number (R0) and the incubation period (α). The incorporation of distinct parameters is shown in the flow diagram (Figure 1).

We accounted for age differences in severe rates, length of recovery, mortality rates, and vaccine protection rates to improve the projection of the reality. Age-specific parameters were included in the model, such as those vaccinated over 60 years old (V_O_) and those vaccinated under 60 years old (V_Y_), as well as vaccine efficacy in preventing severe illness (θ1, θ2, and θ3). We also consider the proportion of susceptible individuals aged 60 and above (ω) and the baseline severity rate of COVID-19 for young (ρ1) and older adult (ρ2) individuals. Our model incorporates the proportion of asymptomatic infections (δ) and the recovery periods for mild (γ), severe for young (γ1), and severe for older adult (γ2) cases. The description of the parameters and values is indicated in Supplementary Table 1.

As of 10 December 2022, the two- and three-dose vaccine uptake in China was 91% and 57%, respectively. Since more than 95% of vaccines administered are inactivated virus vaccines, we assume that all vaccines received are inactivated virus vaccines for their first two doses. It is reported that heterologous booster vaccination would enhance the protection against severe death (23), but the effects of different sequential vaccinations are different. Therefore, we simulate the vaccination effect by inputting parameters revealed by real-world studies to simulate the reality as possible (Table 1).

**Table 1:** Vaccine effectiveness (VE) in preventing Omicron infection.

**Vaccine (combination)**	**Vaccine effectiveness (VE) in preventing Omicron infection**	**Source**	**Vaccine effectiveness (VE) against hospitalization**	**Source**
CoronaVac × 3 (< 60 days after the 3rd dose)	8.6% (95% CI, 5.6–11.5)	Ranzani et al. (24)	86.3% (83.7–88.5)	Alejandro Jara, (25)
CoronaVac × 2 + BNT162b2	56.8% (95% CI, 56.3–57.3)		96.1% (95.3–96.9)	
CoronaVac × 2 + AZD1222	-		97.7% (97.3–98.0)	
BBIBP-CorV × 2 + V-01^*^ (< 60 days after the 3rd dose)	64% (95% CI, 23–83)	Wang et al. (17)		
CoronaVac × 2 + V-01^*^ (< 60 days after the 3rd dose)	39% (95% CI, 3–62)			

V-01^*^ refers to the recombinant SARS-CoV-2 fusion protein vaccine.

To account for the effects of vaccination on the transmission dynamics of the COVID-19 outbreak, we incorporated the vaccination rates of different age groups and the efficacy of vaccine protection against critical diseases. Specifically, we added the rate of protected critical disease θ for booster shots of three different types of vaccines [inactivated(θ_1_), mRNA(θ_2_), and adenovirus-vectorθ_3_], based on the efficacy of three inactivated vaccines. Using this information, we calculated the rate of severe disease after vaccine protection μ as follows:


$$\begin{array}{l} \mu =\rho *\left(1-\text{vaccination proportion}*\;\theta \right), \end{array}$$

where ρ represents the severe rate, and θ represents vaccine effectiveness against severe COVID-19. During the model development process, we incorporated the presence or absence of symptoms in exposed individuals and accounted for the potential challenge of medical resources. Furthermore, we also considered the vaccination status of different age groups by stratifying the population accordingly. Finally, both infected and critical individuals gradually heal and turn into recovered individuals. Thus, our proposed model is the following:


(1) $$\left\{ \begin{array}{l} \frac{dS\left(t\right)}{dt}=\;-\frac{\beta *S\left(t\right)*\left(I_{Y}S\left(t\right)+I_{O}S\left(t\right)+I_{Y}V\left(t\right)+I_{O}V\left(t\right)+A\left(t\right)\right)}{N}\\ \frac{dE\left(t\right)}{dt}=\frac{\beta *S\left(t\right)*\left(I_{Y}S\left(t\right)+I_{O}S\left(t\right)+I_{Y}V\left(t\right)+I_{O}V\left(t\right)+A\left(t\right)\right)}{N}-\alpha *E(t)\\ \frac{dI_{Y}S\left(t\right)}{dt}=\;(1-\delta )*(1-\omega )*(1-\varepsilon )*\alpha *E(t)-(\mu_{1}+\gamma )*I_{Y}S(t)\\ \frac{dI_{O}S\left(t\right)}{dt}=\;(1-\delta )*\omega *(1-\varepsilon )*\alpha *E(t)-(\mu_{2}+\gamma )*I_{O}S(t)\\ \frac{dI_{Y}V\left(t\right)}{dt}=\;(1-\delta )*(1-\omega )*\varepsilon *\alpha *E(t)-(\mu_{1}+\gamma )*I_{Y}V(t)\\ \frac{dI_{O}V\left(t\right)}{dt}=\;(1-\delta )*\omega *\varepsilon *\alpha *E(t)-(\mu_{2}+\gamma )*I_{O}V(t)\\ \frac{dA\left(t\right)}{dt}=\;\delta *\alpha *E(t)-\gamma *A(t)\\ \frac{dH_{Y}\left(t\right)}{dt}=\;\mu_{1}*(I_{Y}S(t)+I_{Y}V(t))-\gamma_{1}*H_{Y}(t)\\ \frac{dH_{O}\left(t\right)}{dt}=\;\mu_{2}*(I_{O}S(t)+I_{O}V(t))-\gamma_{2}*H_{O}(t)\\ \frac{dR\left(t\right)}{dt}=\gamma *(I_{Y}S(t)+I_{O}S(t)+I_{Y}V(t)+I_{O}V(t)+A(t))+\gamma_{1}*H_{Y}(t)+\gamma_{2}*H_{O}(t)\\ \text{S}=\text{ N}-{\text{E}}_{0}-{\text{I}}_{0}-{\text{A}}_{0}-{\text{H}}_{0}-{\text{R}}_{0}\\ \beta =\;R_{t}*\gamma \end{array}\right.$$

To calculate the transmission coefficient β and the effective reproduction number Rt in our model, we utilized the generation-interval-based method proposed by Wallinga J (26).


(2) $$\begin{array}{l} {\text{p}}_{\text{ij}}=\text{w}\left({\text{t}}_{\text{i}}-{\text{t}}_{\text{j}}\right)/\sum_{\text{i}\neq \text{k}}\text{w}\left({\text{t}}_{\text{i}}-{\text{t}}_{\text{k}}\right) \end{array}$$

(3) $$\begin{array}{l} {\text{R}}_{\text{j}}=\sum_{\text{i}}{\text{p}}_{\text{ij}} \end{array}$$

The relative probability of an individual i being infected by an individual j is determined by the generation interval, which accounts for differences in the time between the onset of symptoms in the two cases. This probability can be represented by a probability distribution, which is used to calculate the effective reproduction number Rt in equation (3). The resulting distribution of Rt can be used to estimate the number of future exposed, infected, and removed individuals by inputting it into the modified SEIR model described by equation (1). Furthermore, by utilizing a 95% confidence interval of Rt, we can obtain a distribution of the number of future cases with varying degrees of uncertainty.

Overall, our model provides a robust framework for analyzing the transmission dynamics of the COVID-19 outbreak, while considering important demographic and medical factors that may influence the spread of the disease.

### Effectiveness of NPIs

Hong Kong has experienced the fifth and sixth COVID-19 wave (being Omicron) whereas Shanghai has had one dominated by Omicron BA.2 that led to 2 months of city-wide lockdown. Kathy Leung et al. have categorized NPIs implemented during these waves into four levels and estimated their effectiveness from the associated changes in reproductive number (27). Considering the epidemic during the relaxation of zero COVID-19 is also driven by Omicron, we refer to their experience to quantify the effectiveness of NPIs (Supplementary Table 2).

### Scenario assumptions

We map China's combinations of NPIs and their intensity to the above-mentioned NPI levels. We assume that the NPIs after reopening (7 December 2022) are as effective as Level 1, which would reduce Rt by 15%. A dynamic adjustment of NPIs is anticipated. We assume that the majority of schools were on winter holidays after a month (equivalent to level 2 NPI, which reduces Rt by 44%, Supplementary Table 2) and that 30% of the population had stockpiled drugs and self-isolated due to the government's health education campaign after reopening. The simulation considers the following conditions:

(1) 20,000 omicron-infected individuals were introduced into the Chinese population on 7 December 2022.
(2) The basic reproduction number (R_0_) at the start of the simulation was set to 8.3, and the effective contact rate was 70%.
(3) As of 13 December 2022, the susceptible population in the baseline scenario was 1.4 billion, based on the vaccine efficacy (VE) against infection reported in Table 1.
(4) The VE is set according to the values in Table 1.
(5) The medical capacity at that time was taken into account.
(6) The model was simulated in 120 days.

### Assessment of the overwhelming healthcare resource

According to a report by the National Health Commission of the People's Republic of China (28), there are 33,000 secondary or tertiary hospitals, 19,400 community health centers, and 22,000 fever sentinel sites that operate fever clinics nationwide. Assuming a maximum daily capacity of 150 visits in secondary and higher hospitals and 50 visits in community health and fever sentinel sites, the national maximum capacity for a single day is approximately 7.02 million visits. According to the 2016 National Healthcare Services and Quality Safety Report (29), the average number of respiratory beds in tertiary general hospitals in China is 61.15 and 41.46 in secondary general hospitals. Furthermore, there are 273,000 emergency transition beds, and the capacity of admission beds is 0.908 million.

Considering that COVID-19 patients may be asymptomatic, this group of patients does not pose a burden on the healthcare system. Moreover, mild COVID-19 are recommended to rest at home and take medication on their own, while there was a shortage of medication in the initial period of reopening. We assumed that mild COVID-19 without drug stockpiles and those severe would visit hospitals and take up resources for emergency and inpatient care, respectively. Therefore, we compared the projected number of hospital visits to the daily maximum admissible capacity to assess the overwhelming of healthcare resources.

## Result

### Baseline scenario

The baseline scenario simulated a situation where the majority of schools were on winter holidays (equivalent to level 2 NPI), and 30% of the symptomatic population had stockpiled drugs and therefore isolated themselves at home. The simulation involved the following conditions: (1) 20,000 Omicron-infected individuals were introduced into the Chinese population on 7 December 2022; (2) the basic reproduction number (R0) at the start of the simulation was set to 8.39, effective contact rate is 70% (considering NPIs reached level 2 at the time of reopening); (3) as of 13 December 2022, based on the vaccine efficacy (VE) against infection in Table 1, the current input susceptible population in the baseline scenario is 1.4 billion; (5) the VE against infection and developing severe is reported in Table 1; and (6) the simulation period is 120 days.

**Figure 2:**
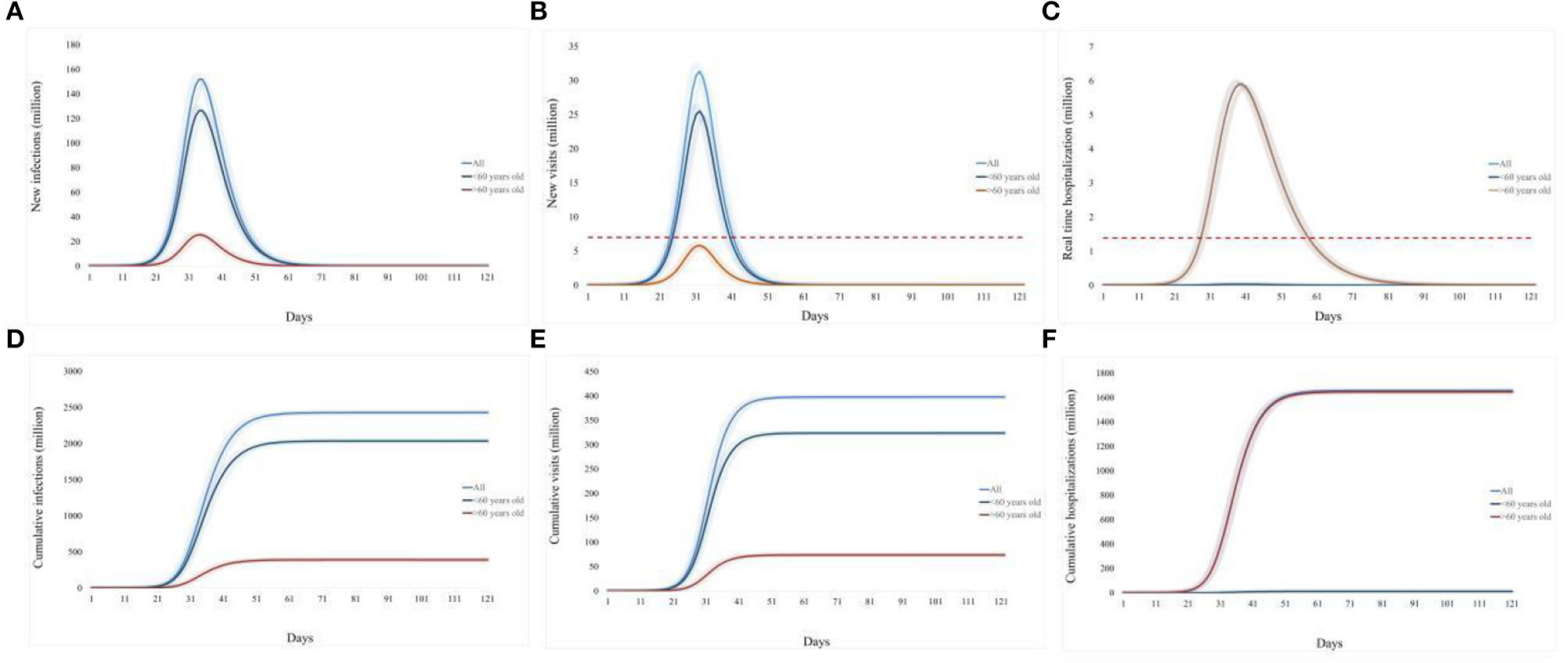
Prediction of the medical resource burden in China's Omicron outbreak under the baseline scenario from 7 December 2022 to 7 March 2023. **(A)**, Daily new infections; **(B)**, daily new emergency department visits; **(C)**, daily existing hospitalizations; **(D)**, cumulative infection over 120 days; **(E)**, cumulative emergency department visits over 120 days; **(F)**, cumulative hospitalizations over 120 days. The light blue curve represents all age groups, the dark blue line represents individuals under 60 years old, and the brown line represents individuals over 60 years old. The red dotted line refers to the existing medical capacity.

The simulated baseline scenario shows that the Omicron variant in China after the reopening on 7 December 2022, could trigger a tsunami of COVID-19 cases under the current epidemic control policy and vaccination rates. During the 120-day simulation period, the epidemic peaks on day 34, with an infection rate of over 1.1 billion (~80% of the population in China) and a single-day peak of 152.13 million infected individuals (Figures 2A, D).

To evaluate the impact of this Omicron epidemic on the national healthcare system, we consider that symptomatic patients without drug stockpiling would attend the emergency department visit, and severe or critical COVID-19 patients would require hospital beds for respiratory illness.

The simulated baseline scenario shows that the Omicron variant in China after the reopening on 7 December 2022, could trigger a tsunami of COVID-19. During the 120-day simulation period, a single-day peak of 152.13 million infected individuals reached on day 34 (Figure 2A). Nationwide, as to the impact on the medical system, the estimated single-day peak of emergency department visits would reach 300 million visits on day 32, 4.4 times the current capacity, and the overwhelming period would last for 18 days (Figure 2B). The daily peak of hospitalization number (5.91 million, day 40) is 6.5 times of admission capacity for respiratory diseases in China, and the bed shortage period is estimated to last 36 days (Figure 2C).

Overall, this epidemic will raise an infection number of over 1.1 billion (80% of the population in China, Figure 2D), and result in 398.02 million emergency department visits (Figure 2E) and 16.58 million hospitalizations (Figure 2F).

### Effectiveness of individual mitigation strategies

**Figure 3:**
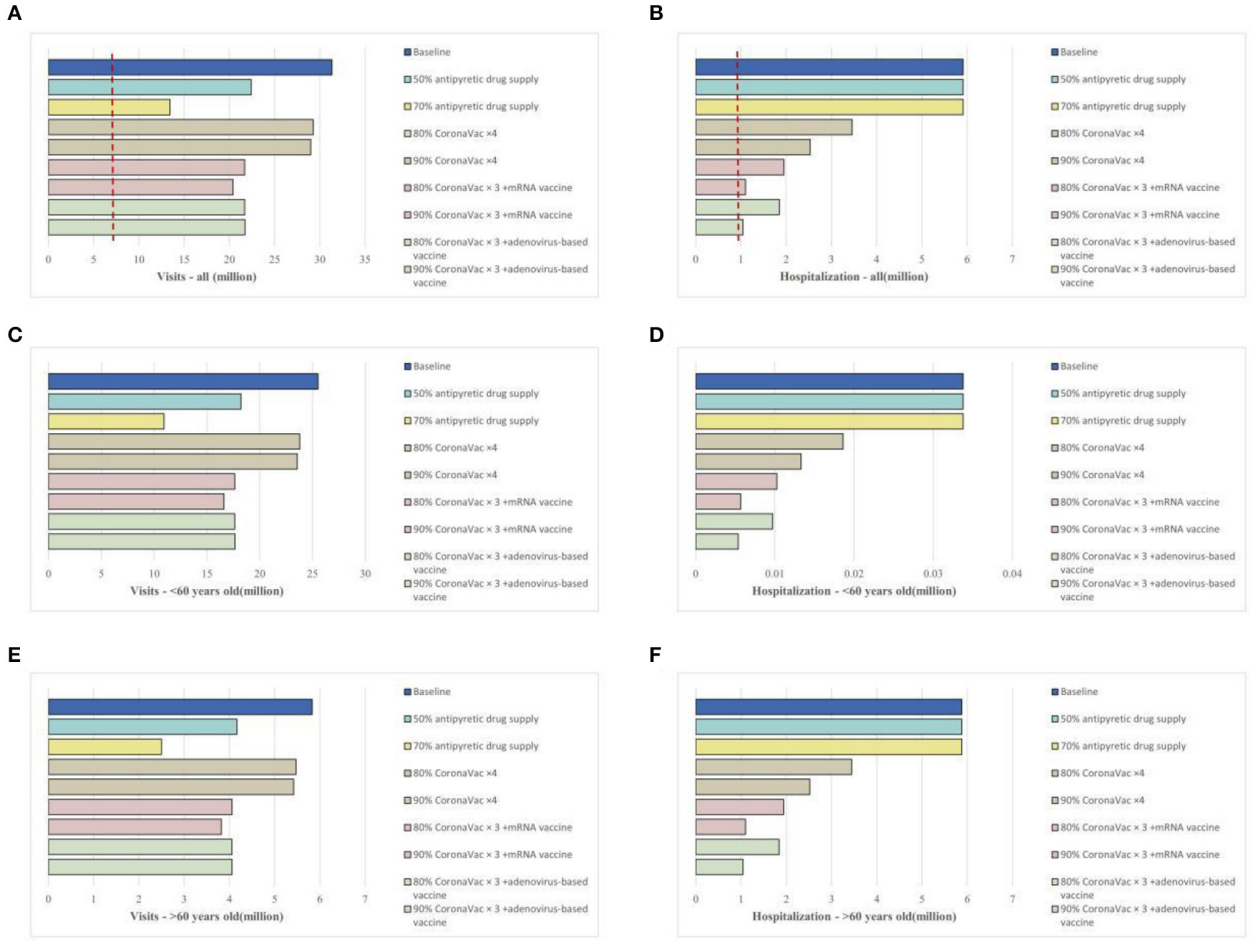
Impact of vaccination strategies or epidemic material reserves on the medical resource burden in China's Omicron outbreak under the baseline scenario. **(A)**, Daily new emergency department visits for all age groups; **(B)**, daily existing hospitalizations for all age groups; **(C)**, daily new emergency department visits for individuals over 60 years old; **(D)**, daily existing hospitalizations for individuals over 60 years old; **(E)**, daily new emergency department visits for individuals under 60 years old; **(F)**, daily existing hospitalizations for individuals under 60 years old.

We study the impact of two strategies to alleviate the COVID-19 burden: (1) vaccine (promoting the fourth dose vaccination coverage with homologous and heterologous boosters) and (2) antipyretic drug stockpiling. In terms of vaccination strategies, increasing the coverage of homologous booster vaccinations (inactivated vaccines) did not significantly reduce the total number of emergency department visits. However, heterologous booster vaccinations (including mRNA and adenovirus-based boosters) significantly reduced the daily number of visits. Compared to the baseline scenario, increasing the fourth dose vaccine rate to 80–90% could reduce the daily visit by 1.4–1.5 times and shorten the peak visit period by 3 days, with similar effects observed in different age groups (Figures 3A, C, E).

Filling the vaccination coverage gaps (i.e., vaccinating all eligible individuals) could effectively reduce hospitalization rates, mainly impacting those over 60 years old. Improving homologous booster vaccinations (inactivated vaccines) at 80% and 90% coverage could reduce hospitalizations by 41.5% and 57.2% and shorten the bed shortage period by 7 and 11 days, respectively. Heterologous booster vaccinations could further reduce hospitalizations, with 80% coverage (mRNA or adenovirus) reducing hospitalizations by 67–68% and shortening the bed shortage period by more than half. With 90% heterologous booster coverage (mRNA or adenovirus), hospitalizations would be reduced by 82%, and there would be no bed shortage (Figures 3B, D, F).

Regarding antipyretic drug supply, we assumed that 50% and 70% of the population could access medications such as antipyretics or antiviral drugs and therefore facilitate at-home isolation when feasible. The results showed (Figure 3A) that this approach could reduce the total emergency department visit number by 1.4–2.3 times and shorten the peak visit period by 6 days. The impact of drug reserves was more concentrated in individuals under 60 years old, with 70% personal drug reserve supply greatly alleviating the pressure on emergency department visits (reducing the number of visits by up to 90%) (Figure 3C). This mitigation measure did not significantly impact the bed burden for respiratory diseases (Figures 3B, D, F).

### Impact of combined mitigation strategies on healthcare resources in China after reopening

**Figure 4:**
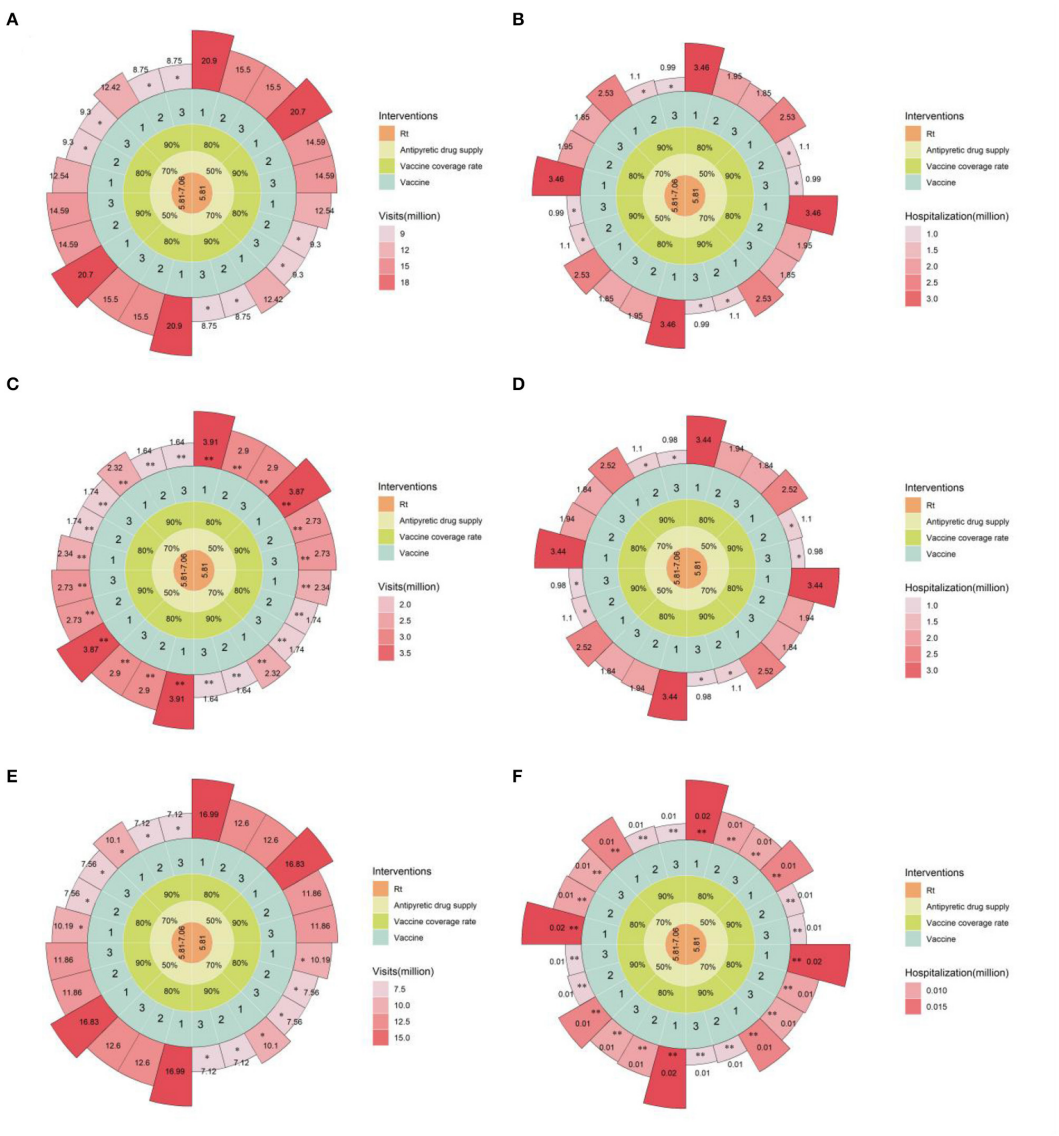
Estimated daily emergency department visits and hospitalizations under combined mitigation strategies in the baseline scenario. **(A)**, Daily new emergency department visits for all age groups; **(B)**, daily existing hospitalizations for all age groups; **(C)**, daily new emergency department visits for individuals over 60 years old; **(D)**, daily existing hospitalizations for individuals over 60 years old; **(E)**, daily new emergency department visits for individuals under 60 years old; **(F)**, daily existing hospitalizations for individuals under 60 years old. **indicates that the available single-day outpatient and inpatient capacity is lower than the existing available outpatient and inpatient capacity in China, *indicates that the available single-day outpatient and inpatient capacity is below 1.5 times the available outpatient and inpatient capacity in China. From the innermost to the outermost concentric circles, the circular Manhattan plot shows the combination of different intervention measures: 1 to 3 for CoronaVac x 4, CoronaVac x 3 + mRNA vaccine, CoronaVac x 3 + adenovirus vaccine booster schemes, respectively; 80 or 90% vaccination coverage in the population after policy liberalization; 50 or 70% personal epidemic material reserves; Rt represents different levels of NPI intensity. Rt = 5.81–7.06 corresponds to the situation where the first 60 days are under Level 2 NPI and the following 60 days under Level 1 NPI, while Rt = 5.81 corresponds to the entire 120-day simulation period being under Level 2 NPI.

Our results showed that relying solely on individual mitigation strategies could not reduce emergency department visits and hospitalization to the current level of national healthcare resource allocation and admission capacity. Here, we evaluated the synergy of several different strategies: increasing vaccine coverage among unvaccinated individuals (including homologous or heterologous vaccination), personal epidemic material reserves, adopting different levels of NPI, and expanding healthcare resource allocation and service capacity by 1.5 times (daily emergency department visits of 0.908 million, converting general outpatient beds to respiratory specialty outpatient beds totaling 1.362 million). By combining different strategies for synergy, as shown in Figure 4, increasing the heterologous booster vaccination rate for older adult to over 90% is a key measure to alleviate the admission burden of emergency department admission (Supplementary Table 3). Furthermore, increasing personal anti-COVID drug reserves to over 70% could significantly reduce emergency department visits. The synergy between different strategies can effectively prevent overburdening the nation's healthcare resource allocation and service capacity, fundamentally alleviating the pressure to treat patients, and laying a solid foundation for the turning point in the COVID-19 epidemic.

## Discussion

Our study projected the number of fever clinic visits and hospitalizations in China after releasing the zero COVID-19 policy. We estimated that the introduction of the Omicron variant would cause substantial surges in fever clinics and hospitalization, and the peak demand would overwhelm the healthcare system with an estimated burden of four times the available capacity. This epidemic is projected to result in 398.02 million fever clinic visits and 16.58 million hospitalizations, and the disruption period on the healthcare system is 18 and 30 days, respectively.

We found antipyretic drug supply and booster vaccination could reduce the burden on emergency visits and hospitalization, respectively. However, considering the current status, they cannot cope with the medical resources overwhelming raised by the existence of the zero COVID-19 policy. Lack of bed capacity, scarcity in drug supplies, and high occupancy rates further increase that burden.

Vaccination is effective in preventing COVID-19 patients from developing severe and death. As of November 2022, 0.89 billion have received three doses of inactivated vaccine in China (30). Our results reveal that the fourth dose of homologous inactivated vaccination and sequential mRNA vaccination introduced could reduce only a modest proportion of fever clinic visits. This indicates the limited effectiveness of 4-dose inactivated homologous vaccination and mRNA sequential vaccination against Omicron infection, which is consistent with the real-world studies in Hong Kong that the short-term effectiveness against Omicron infection of a third or fourth dose of either the mRNA or inactivated vaccine (31). Nevertheless, 4-dose vaccination significantly reduced the number of hospitalizations and the effects were similar between booster vaccine scenarios in inactivated vaccine and mRNA vaccine, which echoes with previous studies (32, 33). In addition, we found that the fourth dose of adenovirus vaccination was significantly reducing the peak both in fever clinic visits and hospitalization. This provides a new strategy for sequential vaccination in response to the co-prevalence of multiple SARS-CoV-2 variants, but monitoring of vaccine protective effects should be enhanced considering the lack of real-world study evidence.

In a questionnaire survey of Omicron infection in Macau after the lift of the zero COVID-19 policy, more than 60% of Omicron-infected patients had symptoms, such as fever (≥37.5C), dry or sore throat, blocked or runny nose, fatigue, headache, muscle soreness, that (32) may require antipyretic and other pharmacological interventions. An adequate supply of antipyretic medications at the onset of peak infection can safeguard the at-home health management of mild and asymptomatic COVID-19 patients, leaving valuable emergency resources for acute and critically ill patients.

We found that expanding the available capacity by 1.5 times would reduce the burden of public health decisions, and how to flexibly increase the admission capacity is the actual problem to be solved. In Singapore, the Public Health Preparedness Clinic (PHPC) system played a critical role in keeping general hospitals free of medical overcrowding after the outbreak. Such a triage treatment enables a large number of patients with mild-to-moderate COVID-19 to be treated in community clinics. In addition, telemedicine is recommended, to, reduce the face-to-face infection and to simplify the consultation, which was also undertaken in England, UK, in 2021 (34). With as many as 10,000 primary hospitals in China, improving and enhancing the level of care in secondary hospitals is a future endeavor that will facilitate the allocation of resources from tertiary hospitals to focus on critical patients and other non-neoconcentric emergencies.

This study has several limitations. First, large-scale nucleic acid screening is no longer centrally organized in China after reopening, so real infection status in the population is not available for validation. To fill this gap, the real-time reproductive number was inferred from the strength of current NPIs, and a 5% range was set to cover as many scenarios as possible. Moreover, we compared the prediction with nationwide monitoring data (Supplementary Figure 1) and found that the differences between the two data in the peak time of fever outpatient visits and hospitalizations were within a week. Second, we assumed that the vaccine effectiveness did not change over time during the projection because the decline of the neutralizing antibody of the fourth dose vaccination is not clear so far.

As antibodies decay and virus mutation, a second peak of SARS-CoV-2 infections may re-emerge in China. We compiled information on COVID-19 outbreaks in 22 developed countries or regions in 2022 (35), finding that the average period between two infection peaks was 149.35 days (median: 161 days), and the average number of new cases in the second peak of infection was 48.61% (median: 41.72%) of the first peak (Supplementary Table 4). As SARS-CoV-2 continues to mutate, ongoing clinical symptom surveillance and vaccine efficacy assessment will help us recognize the transmission risk (36–38). Our study will keep an eye on different durations of the symptoms in subsequent waves and focus on long-time immunogenicity and effectiveness of full vaccination and booster dose of COVID-19 vaccine (based on age and chronic conditions) so that the new round need on the medical system could be timely estimated. Our study provides several suggestions to alleviate the crowding of medical resources due to the above possible infection peak, which helps facilitate early layout and coordinated deployment of prevention and control resources.

## Conclusion

The Omicron epidemic followed the adjustment of COVID-19 policy overload in many local health systems across the country, and the combined effect of vaccination, antipyretic drug supply, triage treatment, and PHSMs could prevent overwhelming medical resources.

## Data availability statement

The raw data supporting the conclusions of this article will be made available by the authors, without undue reservation.

## Author contributions

JL: Conceptualization, Formal analysis, Writing—original draft, Writing—review & editing. RC: Data curation, Writing—original draft, Writing—review & editing. ZL: Methodology, Software, Writing—review & editing. YW: Formal analysis, Writing—original draft. WH: Visualization, Writing—review & editing. RL: Supervision, Writing—review & editing. JS: Resources, Writing—review & editing. QL: Resources, Writing—review & editing. LL: Formal analysis, Writing—review & editing. MZ: Formal analysis, Writing—review & editing. ZC: Funding acquisition, Writing—review & editing. YG: Software, Writing—review & editing. WZ: Resources, Writing—review & editing. TL: Investigation, Writing—review & editing. AO: Data curation, Writing—review & editing. CH: Data curation, Investigation, Visualization, Writing—review & editing.
