# Supplementary material for: Temporary impact on medical system and effectiveness of mitigation strategies after COVID-19 policy adjustment in China: a modeling study
Source: Front Public Health. 2023 Dec 1;11:1259084. doi: 10.3389/fpubh.2023.1259084 (PMC10722892; doi:10.3389/fpubh.2023.1259084)
Supplement: Supplementary file 1 [file Table_1.DOCX]

**Supplementary material**


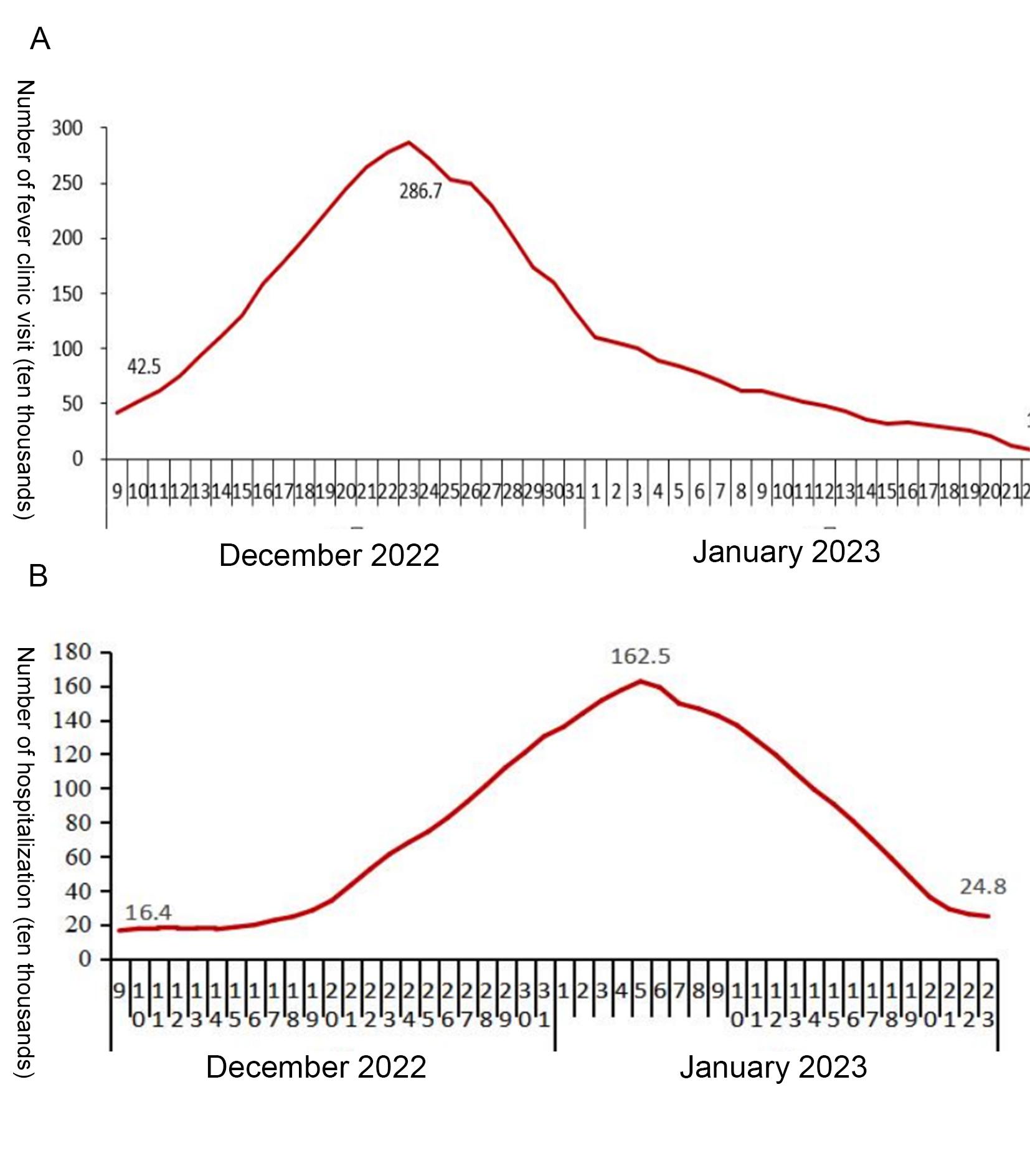


**Supplementary Figure 1. Nationwide surveillance data of COVID-19.** A. Changes in the positive rate of nucleic acid testing nationwide number of COVID-19 patients hospitalized. Data source: Chinese Centre for Disease Control and Prevention.

**Supplement Table 1. Parameter and references**

| Type | Parameter | Label | Value | References |
| --- | --- | --- | --- | --- |
| Demographic parameter | Number of total individuals | S | 1411778724 | https://www.gov.cn/guoqing/2021-05/13/content_5606149.htm |
|  | Proportion of booster vaccinations over 60 | V_O_ | 69.8% | https://finance.china.com.cn/news/20221214/5914896.shtml |
|  | Proportion of booster vaccinations under 60 | V_Y_ | 55% |  |
|  | Proportion of susceptible individuals over 60 | ω | 18.6% |  |
| Epidemiological parameter | Basic reproduction number | R0 | 8.3 | Kathy Leung , et al. MedRxiv. 2022 |
|  | Incubation period | α | 3.5 | Jansen L, et al. 2021. |
|  | Proportion of asymptomatic infected individuals | $\delta$ | 32.4% | Weijing Shang, et al.Vaccines (Basel). 2022. |
| Parameter on disease progression | Severe rate of young people among infected individuals | $\rho_{1}$ | 0.011% | Jun Cai  et al. 2022 |
|  | Severe rate of old people among infected individuals | $\rho_{2}$ | 7.98% | Jun Cai  et al. 2022 |
|  | Recovery period for mild/asymptomatic infected individuals | $\gamma$ | 4.4 | Menni C, et al. Lancet. 2022 |
|  | Recovery period of young critical individuals | $\gamma_{1}$ | 6 | Jun Cai  et al. 2022 |
|  | Recovery period of old critical individuals | $\gamma_{2}$ | 8 | Guanzhu Lu et al. 2022 |
| Vaccine relevant parameter values | Effectiveness against severe COVID-19 of booster inactivated vaccine | $\theta_{1}$ | 86.3% | Alejandro Jara, et al. 2022 |
|  | Effectiveness against severe COVID-19 of booster mRNA vaccine | $\theta_{2}$ | 96.1% |  |
|  | Effectiveness against severe COVID-19 of booster adenovirus-vector vaccine | $\theta_{3}$ | 97.7% |  |

**Supplement Table 2. Effectiveness of NPIs during the previous waves of COVID**

| **Second~ Fourth wave in Hong Kong** | | |
| --- | --- | --- |
| **Level** | **NPI** | **Reduction in Rt** |
| leve1 | Wear masks in public places and pay attention to personal hygiene | 15% |
| level2 | On the basis of level, closure of some schools, tightened indoor amenities control. | 30-47% |
| level3 | On the basis of level, gathering in groups of 4 only, closure of all indoor amenities, no dine-in after 6 pm, closure of all schools, civil servants work-from-home | 52-76% |
| level4 | On the basis of level, tightened measures in restaurants and  indoor amenities, closure of outdoor and indoor amenities | 78-91%* (83-96) |
| **Italy (the summer of 2020) (**Liu et al., 2022**)** | | |
|  | **NPI** | **Increase in Rt** |
|  | The schools reopened in mid-September 2020 | 35% |
| **Hong Kong (January 2020 to March 2021)** (Cowling et al., 2020) | | |
|  | **NPI** | **Reduction in Rt** |
|  | closure of school | 44% |

**Supplement Table 3. The different combinations of scenarios for existing peak inpatient admissions and single-day peak emergency department visits(million)**

| Combined scenario | | Rt | | Antipyretic drug supply | | Vaccine | <60 years old - hospitalization | | | >60 years old - hospitalization | | all - hospitalization | | <60 years old - visits | | >60 years old - visits | | all - visits |
| --- | --- | --- | --- | --- | --- | --- | --- | --- | --- | --- | --- | --- | --- | --- | --- | --- | --- | --- |
| 1 | | 5.81-7.06 | | 50% | | 80%(CoronaVac×4） | 0.0185999 | | | 3.4426586 | | 3.4612585 | | 16.9890274 | | 3.9076853 | | 20.8967126 |
| 2 | | 5.81-7.06 | | 50% | | 80%(CoronaVac×3+mRNAvaccine） | 0.0102732 | | | 1.9400227 | | 1.950199 | | 12.5987495 | | 2.8978674 | | 15.4966169 |
| 3 | | 5.81-7.06 | | 50% | | 80%(CoronaVac×3+adenovirus-basedvaccine） | 0.0097053 | | | 1.8390514 | | 1.8486642 | | 12.6000428 | | 2.8981648 | | 15.4982076 |
| 4 | | 5.81-7.06 | | 50% | | 90%(CoronaVac×4） | 0.0132994 | | | 2.5177856 | | 2.5310851 | | 16.8321058 | | 3.8715914 | | 20.7036972 |
| 5 | | 5.81-7.06 | | 50% | | 90%(CoronaVac×3+mRNAvaccine） | 0.0056634 | | | 1.0972041 | | 1.1028676 | | 11.8584984 | | 2.7276005 | | 14.5860989 |
| 6 | | 5.81-7.06 | | 50% | | 90%（CoronaVac×3+adenovirus-basedvaccine） | 0.0050602 | | | 0.9841058 | | 0.989166 | | 11.8627804 | | 2.7285854 | | 14.5913659 |
| 7 | | 5.81-7.06 | | 70% | | 80%（CoronaVac×4） | 0.0185999 | | | 3.4426586 | | 3.4612585 | | 10.1934164 | | 2.3446112 | | 12.5380276 |
| 8 | | 5.81-7.06 | | 70% | | 80%（CoronaVac×3+mRNAvaccine） | 0.0102732 | | | 1.9400227 | | 1.950199 | | 7.5592497 | | 1.7387204 | | 9.2979701 |
| 9 | | 5.81-7.06 | | 70% | | 80%（CoronaVac×3+adenovirus-basedvaccine） | 0.0097053 | | | 1.8390514 | | 1.8486642 | | 7.5600257 | | 1.7388989 | | 9.2989246 |
| 10 | | 5.81-7.06 | | 70% | | 90%（CoronaVac×4） | 0.0132994 | | | 2.5177856 | | 2.5310851 | | 10.0992635 | | 2.3229548 | | 12.4222183 |
| 11 | | 5.81-7.06 | | 70% | | 90%（CoronaVac×3+mRNAvaccine） | 0.0056634 | | | 1.0972041 | | 1.1028676 | | 7.115099 | | 1.6365603 | | 8.7516593 |
| 12 | | 5.81-7.06 | 70% | 90%（CoronaVac×3+adenovirus-basedvaccine） | | | 0.0050602 | 0.9841058 | | 0.989166 | | 7.1176683 | | 1.6371512 | | 8.7548195 | |  |
| 13 | | 5.81 | 50% | 80%（CoronaVac×4） | | | 0.0185999 | 3.4426586 | | 3.4612585 | | 16.9890274 | | 3.9076853 | | 20.8967126 | |  |
| 14 | | 5.81 | 50% | 80%（CoronaVac×3+mRNAvaccine） | | | 0.0102732 | 1.9400227 | | 1.950199 | | 12.5987495 | | 2.8978674 | | 15.4966169 | |  |
| 15 | | 5.81 | 50% | 80%（CoronaVac×3+adenovirus-basedvaccine） | | | 0.0097053 | 1.8390514 | | 1.8486642 | | 12.6000428 | | 2.8981648 | | 15.4982076 | |  |
| 16 | | 5.81 | 50% | 90%（CoronaVac×4） | | | 0.0132994 | 2.5177856 | | 2.5310851 | | 16.8321058 | | 3.8715914 | | 20.7036972 | |  |
| 17 | | 5.81 | 50% | 90%（CoronaVac×3+mRNAvaccine） | | | 0.0056634 | 1.0972041 | | 1.1028676 | | 11.8584984 | | 2.7276005 | | 14.5860989 | |  |
| 18 | | 5.81 | 50% | 90%（CoronaVac×3+adenovirus-basedvaccine） | | | 0.0050602 | 0.9841058 | | 0.989166 | | 11.8627804 | | 2.7285854 | | 14.5913659 | |  |
| 19 | | 5.81 | 70% | 80%（CoronaVac×4） | | | 0.0185999 | 3.4426586 | | 3.4612585 | | 10.1934164 | | 2.3446112 | | 12.5380276 | |  |
| 20 | | 5.81 | 70% | 80%（CoronaVac×3+mRNAvaccine） | | | 0.0102732 | 1.9400227 | | 1.950199 | | 7.5592497 | | 1.7387204 | | 9.2979701 | |  |
| 21 | | 5.81 | 70% | 80%（CoronaVac×3+adenovirus-basedvaccine） | | | 0.0097053 | 1.8390514 | | 1.8486642 | | 7.5600257 | | 1.7388989 | | 9.2989246 | |  |
| 22 | | 5.81 | 70% | 90%（CoronaVac×4） | | | 0.0132994 | 2.5177856 | | 2.5310851 | | 10.0992635 | | 2.3229548 | | 12.4222183 | |  |
| 23 | | 5.81 | 70% | 90%（CoronaVac×3+mRNAvaccine） | | | 0.0056634 | 1.0972041 | | 1.1028676 | | 7.115099 | | 1.6365603 | | 8.7516593 | |  |
| 24 | | 5.81 | 70% | 90%（CoronaVac×3+adenovirus-basedvaccine） | | | 0.0050602 | 0.9841058 | | 0.989166 | | 7.1176683 | | 1.6371512 | | 8.7548195 | |  |

**Supplementary material Table 4. Analysis on COVID-19 infection peak in 22 countries/areas**

|  | **1^st^ infection peak date** | **2^nd^ infection peak date** | **Days between the 1st and the 2nd infection peak** | **New confirmed cases of 1^st^ infection peak (7 days average)** | **New confirmed cases of 2^nd^ infection peak (7 days average)** | **Infection peak ratio (Infection peak of the 2nd wave over the 1st wave)** |
| --- | --- | --- | --- | --- | --- | --- |
| **World** | 2022/1/24 | 2022/7/24 | 181 | 431.259 | 139.412 | 32.33% |
| **Australia** | 2022/1/13 | 2022/4/2 | 79 | 4172.098 | 2188.637 | 52.46% |
| **Brazil** | 2022/1/29 | 2022/7/3 | 155 | 878.844 | 310.017 | 35.28% |
| **Canada** | 2022/1/10 | 2022/4/19 | 99 | 1078.537 | 449.94 | 41.72% |
| **France** | 2022/1/25 | 2022/4/1 | 66 | 5405.375 | 2073.144 | 38.35% |
| **Germany** | 2022/3/24 | 2022/7/20 | 118 | 2752.604 | 1176.477 | 42.74% |
| **Japan** | 2022/2/9 | 2022/8/24 | 196 | 762.817 | 1830.996 | 240.03% |
| **Portugal** | 2022/1/31 | 2022/5/20 | 109 | 6044.62 | 2606.265 | 43.12% |
| **Russia** | 2022/2/15 | 2022/9/21 | 218 | 1304.043 | 370.653 | 28.42% |
| **Singapore** | 2022/2/27 | 2022/7/19 | 142 | 3249.994 | 1908.024 | 58.71% |
| **South Africa** | 2022/1/5 | 2022/5/11 | 126 | 145.834 | 128.315 | 87.99% |
| **South Korea** | 2022/3/17 | 2022/8/23 | 159 | 7816.101 | 2497.374 | 31.95% |
| **United Kingdom** | 2022/1/5 | 2022/3/21 | 75 | 2698.919 | 1308.783 | 48.49% |
| **United States** | 2022/1/14 | 2022/7/17 | 184 | 2381.638 | 401.752 | 16.87% |
| **Italy** | 2022/1/14 | 2022/7/14 | 181 | 3079.768 | 1767.325 | 57.39% |
| **Saudi Arabia** | 2022/1/20 | 2022/7/2 | 163 | 154.135 | 31.288 | 20.30% |
| **India** | 2022/1/25 | 2022/7/22 | 178 | 220.144 | 13.952 | 6.34% |
| **Indonesia** | 2022/2/20 | 2022/8/9 | 170 | 202.087 | 20.253 | 10.02% |
| **Mexico** | 2022/1/25 | 2022/7/15 | 171 | 386.827 | 250.103 | 64.66% |
| **Turkey** | 2022/2/8 | 2022/8/6 | 179 | 1211.318 | 680.163 | 56.15% |
| **European Union** | 2022/1/31 | 2022/7/11 | 161 | 2803.955 | 930.676 | 33.19% |
| **Hong Kong** | 2022/3/4 | 2022/9/8 | 188 | 8839.03 | 1367.535 | 15.47% |
| **Taiwan** | 2022/5/25 | 2022/10/9 | 137 | 3451.276 | 1936.16 | 56.10% |
| **Total**  **[Mean, (Min, Max)]** |  |  | 149.35  (66,218) |  |  | 48.61%  (240.03%) |
